# Early Developmental Regulation of Tyrosine Decarboxylase in Olive Fruits and Its Impact on Virgin Olive Oil Phenolics

**DOI:** 10.1021/acs.jafc.5c17310

**Published:** 2026-04-28

**Authors:** Pilar Luaces, Rosario Sánchez, Jesús Expósito, Ana G. Pérez, Carlos Sanz

**Affiliations:** Department of Biochemistry and Molecular Biology of Plant Products, 54444 Instituto de la Grasa, Spanish National Research Council (CSIC), 41013 Seville, Spain

**Keywords:** *Olea europaea*, hydroxytyrosol, tyrosol, tyrosine decarboxylase, olive fruit, phenolics, virgin olive oil

## Abstract

Virgin olive oil (VOO), a hallmark of the Mediterranean diet, owes its health benefits largely to phenolic compounds, particularly secoiridoid derivatives containing tyrosol (Ty) and hydroxytyrosol (HTy). This study investigates the first step in Ty and HTy biosynthesis, catalyzed by tyrosine decarboxylase (TDC), during early fruit development and ripening. We analyzed the expression of two *TDC* genes, *OeTDC1* and *OeTDC2*. Results indicate that neither gene contributes to Ty or HTy synthesis during ripening. In contrast, both genes are strongly induced during the active cell division phase within the first 3 weeks after flowering, coinciding with a sharp increase in Ty and HTy derivatives. Among them, *OeTDC1* appears to play the primary role among the *TDC* genes in this pathway, highlighting its importance in determining the content of Ty and HTy derivatives in the olive fruit and, ultimately, VOO quality.

## Graphical abstract

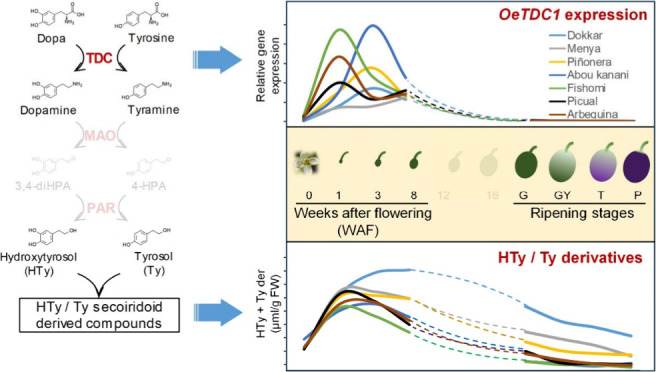

## Introduction

Virgin olive oil (VOO) is a key component of the Mediterranean diet and its health benefits are largely attributed to its phenolic fraction, which contributes to antioxidant activity, flavor, and stability.^1–7^ These phenolics originate from secoiridoid glycosides in olive fruit, primarily oleuropein and ligstroside, which contain hydroxytyrosol (HTy) and tyrosol (Ty) residues.^8,9^ During oil extraction, enzymatic hydrolysis of these glycosides generates the major phenolic compounds found in VOO.^10^ Ty and HTy are synthesized from tyrosine and 3,4-dihydroxyphenylalanine (Dopa) through reactions involving decarboxylation, oxidative deamination, and aldehyde reduction.^11–14^ Tyrosine decarboxylases (TDC; EC 4.1.1.25) catalyze the first step of this pathway and belong to the pyridoxal 5′-phosphate-dependent aromatic amino acid decarboxylase (AAAD) gene family, which plays a central role in the biosynthesis of specialized metabolites.^15^ In olive (*Olea europaea* L.), TDC activity was linked to the formation of Ty and HTy derivatives, which are precursors of secoiridoid phenolics in fruit and oil.^16^ We demonstrated recently the presence of *TDC* genes in olive and characterized the catalytic properties of two *TDC* isoforms involved in Ty and HTy biosynthesis.^17^ However, the relationship between *TDC* gene expression and phenolic accumulation during fruit ripening remains unclear. Understanding this link is essential because, as mentioned above, Ty and HTy are precursors of secoiridoid glycosides that define the phenolic profile of olive fruit and, consequently, VOO quality.

Olive fruit consists of three main tissues, the endocarp, mesocarp, and epicarp, each following distinct growth and differentiation patterns during fruit development. Rosati et al.^18–20^ reported that ovary cell number and size at bloom strongly correlate with cultivar fruit size, which depends on cell division and expansion occurring immediately after bloom until endocarp lignification (pit hardening), followed by cell expansion after pit hardening. Previous studies indicate that some cell division persists at a reduced rate throughout development.^21–23^ More recently, Camarero et al.^24^ provided a detailed characterization of early fruit development in cultivar “Picual” using flow cytometry to assess cell division and expansion phases, cell area, ploidy, and mitotic activity. Their findings show that cell division continues until 3 weeks after flowering (WAF), with the highest proportion of tetraploid cells at 2 WAF, revealing intense endoreduplication immediately after fertilization, a hallmark of cell differentiation, accelerated growth, and increased protein synthesis. Between 3 and 4 WAF, rapid cell expansion occurs, with the expansion rate doubling until 6 WAF before pit hardening. This six week proliferation period mirrors patterns observed in most plant species. Typically, mesocarp cell division accounts for 15–20% of total fruit growth,^25^ except in certain berries, which complete division before anthesis, and avocado, where division persists until ripening.^26^ During this active division phase, Camarero et al.^24^ identified genes sequentially inducing cell wall biosynthesis and remodelling, along with higher expression of cell division-related genes compared to those associated with cell expansion. In line with these observations, our data from the cell expansion phase before pit hardening (3–8 WAF) in different olive cultivars, regarding Ty and HTy derivative content and *TDC* gene,^17^ suggest that the early stages after fruit set may represent a critical window for phenolic biosynthesis, although *TDC* expression dynamics during this period remain unexplored.

The aim of this study was to deepen our understanding of the early growth and ripening processes of olives in relation to the synthesis of Ty and HTy, basic components of the main phenolics in both the fruit and the oil. To this end, the first objective was to analyze *TDC* gene expression levels during the initial stages after fruit set and to assess their correlation with phenolic accumulation in the fruit. The second objective was to determine whether the expression of *TDC* genes is associated with the phenolic content and profile of the olive during fruit ripening and to evaluate its potential impact on the phenolic composition of VOO.

## Materials and Methods

### Reagents

All reagents used for extraction and subsequent analyses were obtained primarily from Sigma-Aldrich (St. Louis, MO, USA). Phenolic standards were sourced from Extrasynthese (Genay, France), including oleuropein (≥98%), verbascoside (≥98%), tyrosol-O-glucoside (≥98%), hydroxytyrosol (≥98%), and tyrosol (≥98%), while ligstroside (≥98%) was provided by ChemFaces (Wuhan, China). Noncommercial compounds such as demethyloleuropein, hydroxytyrosol-glucoside, and major secoiridoid derivatives were isolated from olive leaves, fruits, and oils using solid-phase extraction (SPE) with C18 cartridges (Supelco, Bellefonte, PA, USA) followed by preparative high-performance liquid chromatography (HPLC). HPLC-grade methanol and acetonitrile were supplied by Panreac (Barcelona, Spain). All solutions were prepared with ultrapure water produced by a Milli-Q purification system (Millipore, MA, USA).

### Plant Material

Seven olive cultivars (*Olea europaea* L.), “Dokkar”, “Menya”, “Piñonera”, “Picual”, “Arbequina”, “Fishomi”, and “Abou kanani”, were selected for their contrasting phenolic profiles in fruit and oil.^27^ These cultivars belong to the genetically diverse Core-36 collection maintained at the World Olive Germplasm Bank (IFAPA Alameda del Obispo, Cordoba, Spain).^9^ Two trees per cultivar were sampled from experimental orchards of the Instituto de la Grasa, grown under a 5 × 6 m spacing system with drip irrigation and fertigation from flowering to full ripening. Sampling was performed manually at anthesis and up to 8 weeks after flowering (WAF). Additionally, fruit samples were collected at different ripening stages: G (dark green), GY (green-yellow), T (turning, ∼50% color), and P (purple, fully colored with white mesocarp). Flowers and fruits (20–50 per sample) were taken for both phenolic analysis and RNA extraction, for which they were previously frozen in liquid nitrogen and stored at −80 °C.

### Olive Oil Extraction

Virgin olive oil was extracted from olive fruits using an Abencor extraction system (Comercial Abengoa, S.A., Seville, Spain), which consisted of a stainless steel hammer mill rotating at 3000 rpm equipped with a 5 mm sieve, a mixer operating at 28 °C for 30 min, and a basket centrifuge operating at 3500 rpm for 1 min. Once decanted and filtered with paper, the oils were stored in a nitrogen atmosphere at 4 °C until the extraction of the phenolic compounds.

### Extraction and Analysis of Phenolic Compounds

Phenolic compounds were extracted from fruits following a previously established protocol by García-Rodríguez et al.^28^ Thin longitudinal slices of mesocarp tissue were excised from ripening fruits and incubated at 4 °C for 72 h in dimethyl sulfoxide (6 mL/g of FW) containing syringic acid (24 mg/mL) as an internal standard. Flowers and small fruits from early developmental stages were processed whole. Extracts were filtered through 0.45 μm nylon membranes and stored at −20 °C until HPLC analysis.

VOO phenolic compounds were isolated by solid phase extraction (SPE) on a diol-bonded phase cartridge (Supelco, Bellefonte, PA).^27^ A standard solution (0.5 mL) consisting of *p*-hydroxyphenyl-acetic acid (120 μg/mL) and *o*-coumaric acid (10 μg/mL) in methanol was added to each oil sample (2.5 g) before extraction.

Both fruit and oil phenolic extracts were analyzed as described by García-Rodríguez et al.^28^ using a Beckman Coulter system equipped with a diode array detector and a Superspher RP 18 column (4.6 mm inner diameter x 250 mm, particle size 4 μm, Dr. Maisch GmbH, Ammerbuch, Germany) at 35 °C. Detection was carried out at 280 and 335 nm, and quantification was based on calibration curves and the internal standard. Tentative compound identification by retention time and UV–vis spectra was confirmed by HPLC/ESI-qTOF-HRMS using a Dionex Ultimate 3000 RS UHPLC system (Thermo Fisher Scientific, USA) coupled to a micrOTOF-QII high-resolution time-of-flight mass spectrometer (Bruker Daltonics, Germany) with electrospray ionization.

Fruit phenolics derived from Ty and HTy were classified into two main groups: tyrosol derivatives (Ty-Der), including tyrosol-1-glucoside, ligstroside, and *p*-HPEA-EA (ligstroside aglycone); and hydroxytyrosol derivatives (HTy-Der), comprising hydroxytyrosol-1-glucoside, verbascoside, oleuropein, demethyloleuropein, and 3,4-DHPEA-EA (oleuropein aglycone).

Oil phenolics derived from Ty and HTy were grouped into two main groups: Ty-Der, containing Ty, *p*-HPEA-DEA (oleocanthal), and ligstroside aglycone; and HTy-Der, including HTy, HTy acetate, 3,4-DHPEA-DEA (oleacein), and oleuropein aglycone.

### cDNA Library Construction

cDNA libraries were generated from samples of the seven cultivars collected at different developmental and ripening stages. Total RNA was extracted from three biological replicates per cultivar/stage using the Spectrum Plant Total RNA kit (STRN250, Sigma-Aldrich, USA). RNA concentration and integrity were assessed by Nanodrop spectrophotometry (Thermo Scientific, USA). First-strand cDNA synthesis was performed using the Ready-To-Go You-Prime First-Strand Beads kit (Cytiva, UK) and Oligo­(dT)­18 primers (Thermo Scientific, USA).

### Gene Expression Analysis

Expression of olive *TDC* genes was analyzed by RT-qPCR on a CFX96 Touch System (Bio-Rad, USA) using cDNA libraries, gene-specific primers for *OeTDC1* and *OeTDC2* (Table 1), and SYBR Green I (SsoAdvanced Universal SYBR Green Supermix, Bio-Rad). The thermal profile consisted of an initial denaturation at 95 °C for 30 s, followed by 40 cycles of 95 °C for 15 s, annealing at 51 °C for *OeTDC1* and 54 °C for *OeTDC2* for 15 s, and extension at 60 °C for 15 s. Relative expression was calculated using the Pfaffl method implemented in Bio-Rad CFX Maestro 1.0 software.^29^ Primer efficiencies were determined through serial cDNA dilutions. Olive genes were selected as reference genes according to previous studies^17^ and validated in our samples using the GeNorm algorithm integrated in the BioRad CFX Maestro 1.0 software. Reference genes included *Ser/Thr phosphatase 2A* (*OePP2A*), *glyceraldehyde-3-phosphate dehydrogenase* (*OeGAPDH*), and *elongation factor-1-alpha* (*OeEF1α*) (annotation numbers OE6A097517, OE6A105640, and OE6A045598; https://denovo.cnag.cat/olive_data). Primer sequences for reference genes are listed in Table 1. Three biological and two technical replicates were analyzed per sample.

**Table 1:** Oligonucleotides Used for RT-qPCR

Name	Sequence (5′-3′)	Amplicon size (bp)
q*OeTDC1*-F	ACCCT­TACAG­AAAAC­AGGCA	
q*OeTDC1*-R	TTTTT­GTCCA­TCTTC­ATCTTC	106
q*OeTDC2*-F	ACCCT­TACAG­AAAAC­AGGCA	
q*OeTDC2*-R	CGCAA­ACTAA­AATGG­ACAAA­AGCA	153
q*OeEF1α*-F	TGCTC­TATCT­GGATT­GCCATT	
q*OeEF1α*-R	TCAAA­TGCCA­CCATG­ACTTC	107
q*OeGAPDH*-F	TGAGA­TGCTG­CACAA­TGGTT	
q*OeGAPDH*-R	CACGA­TAGGC­TTACG­CAACA	131
q*OePP2A*-F	CTCGC­CTGAA­AACGA­AAGAC	
q*OePP2A*-R	CACAA­AGCAG­ACCAA­AACCA	194

### Statistical Analysis

Statistical analysis was performed using Excel 2016 and STATISTICA 8.0 (Statsoft Inc., Tulsa, OK).The expression of the olive *TDC* genes was evaluated using Tukey’s test with a significance level of *p* ≤ 0.05. Pearson’s correlation coefficients were calculated to assess the relationships between olive TDC gene expression and the content of tyrosol and hydroxytyrosol derivatives in olive fruit and oil.

## Results and Discussion

According to previous studies, phenolic compound accumulation during olive fruit development occurs predominantly in the early stages,^27^ when most mesocarp and endocarp cells are formed.^21,22^ From this point until ripening, substantial cell expansion occurs in the mesocarp, including additional cell production with cultivar-dependent intensity according to different authors.^21,22^ More recently, Camarero et al.^24^ observed that cell division extends up to 3 WAF, a period characterized by intense endoreduplication. Between 3 and 4 WAF, a strong expansion of the cells occurs, continuing with less intensity until pit hardening initially and then until ripening. Coinciding with the increased expression of genes related to cell division reported by Camarero et al.,^24^ we determined whether the high levels of phenolic compounds observed in olive fruits around pit hardening, as reported in previous studies,^27^ are already present in the flower or are synthesized *de novo* during the cell division phase (1–3 WAF). We analyzed the content and profile of Ty and HTy derivatives both in the flowers (0 WAF) and during this active division stage, before postmitotic cell expansion and pit hardening (3–8 WAF) (Figure 1, Table 2 and Table S1 in Supporting Information).

**Figure 1:**
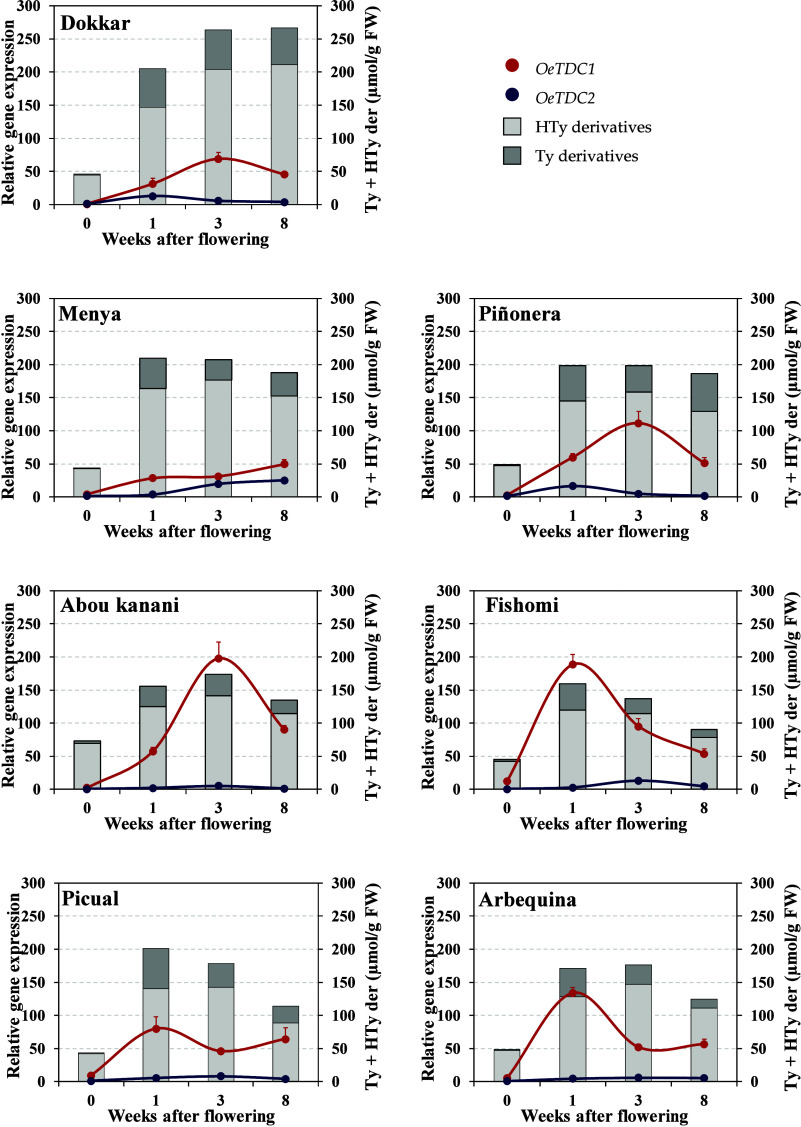
Relative expression levels of olive *TDC* genes during the early olive fruit development. Relative gene expression levels were scaled using as calibrator the expression of the *OeTDC2* gene in the “Dokkar” cultivar at stage T in Figure 2 (control = 1). Data are mean ± SD of three biological and two technical replicates for each sample. Content of derivatives of tyrosol (Ty) and hydroxytyrosol (Hty) (μmol/g FW) were represented according to Table 2.

**Table 2:** Content (μmol/g FW) of the Main Classes of Phenolic Compounds (Hty-Der, Derivatives of Hydroxytyrosol; Ty-Der, Derivatives of Tyrosol; Total, Sum of Hty-Der and Ty-Der) in Olive Fruits of Seven Cultivars during Early Fruit Development from Anthesis (0 Weeks After Flowering, WAF) to 8 WAF^a^

Cultivar	WAF	HTy-Der	Ty-Der	Total
Dokkar	0	44.54 ± 4.78	1.67 ± 0.09	46.22 ± 4.87
	1	146.50 ± 13.30	58.37 ± 4.27	204.87 ± 17.57
	3	203.32 ± 3.33	60.36 ± 2.26	263.69 ± 5.59
	8	211.25 ± 3.70	55.57 ± 0.97	266.82 ± 4.66
				
Menya	0	42.75 ± 0.65	1.18 ± 0.10	43.94 ± 0.55
	1	164.04 ± 3.10	45.40 ± 0.71	209.44 ± 3.81
	3	176.31 ± 10.40	31.03 ± 3.52	207.34 ± 13.92
	8	152.71 ± 4.04	35.50 ± 0.90	188.22 ± 4.93
				
Piñonera	0	47.38 ± 5.56	1.31 ± 0.26	48.69 ± 5.82
	1	145.05 ± 0.33	53.04 ± 6.90	198.09 ± 7.23
	3	158.33 ± 2.31	39.85 ± 0.48	198.18 ± 1.83
	8	129.45 ± 2.92	57.03 ± 0.05	186.48 ± 2.97
				
Abou kanani	0	69.48 ± 3.25	3.30 ± 0.32	72.78 ± 3.58
	1	125.08 ± 4.17	30.67 ± 0.50	155.75 ± 4.67
	3	141.55 ± 3.54	32.15 ± 0.10	173.70 ± 3.45
	8	114.62 ± 0.60	19.73 ± 0.07	134.35 ± 0.67
				
Fishomi	0	41.86 ± 5.49	3.38 ± 0.17	45.23 ± 5.66
	1	119.81 ± 12.76	39.89 ± 6.03	159.70 ± 18.80
	3	114.45 ± 0.58	22.74 ± 0.08	137.18 ± 0.50
	8	78.16 ± 1.92	12.11 ± 0.15	90.27 ± 2.07
				
Picual	0	41.56 ± 4.09	2.03 ± 0.00	43.58 ± 4.09
	1	140.56 ± 8.47	60.72 ± 0.44	201.28 ± 8.04
	3	142.54 ± 9.82	36.42 ± 3.07	178.97 ± 12.89
	8	88.51 ± 2.84	25.52 ± 0.27	114.03 ± 3.11
				
Arbequina	0	47.02 ± 4.66	1.79 ± 0.75	48.81 ± 5.41
	1	128.02 ± 1.95	43.23 ± 3.42	171.26 ± 1.46
	3	147.24 ± 8.11	29.14 ± 2.11	176.38 ± 10.22
	8	111.13 ± 2.14	13.69 ± 0.38	124.82 ± 2.53

a The content of the main individual phenolics can be found in the Supporting Information (Table S1).

Olive flowers exhibited relatively high levels of Ty and HTy derivatives, averaging 50 μmol/g FW across all cultivars studied, except for “Abou kanani”, which showed a significantly higher concentration (73 μmol/g FW). Notably, the proportion of HTy derivatives in flowers was higher (96%) than the average found in fruits during development and ripening, which typically averages around 82% of the total Ty and HTy derivatives. Following ovary fertilization, the synthesis of these compounds accelerates, reaching a peak concentration within 3 weeks. After this point, an apparent decrease in phenolic concentration per fresh weight occurs, except in cultivar “Dokkar”, where the decline is delayed until after 8 WAF.^27^ In reality, this trend largely reflects dilution caused by mesocarp cell expansion and increased cell mass rather than a reduction in absolute phenolic accumulation. When expressed per fruit unit, Ty and HTy derivatives continue to accumulate slowly until just before ripening (Figure S1, Supporting Information). High phenolic levels during fruit development play a defensive role in protecting the fruit when it is most vulnerable to herbivore attacks, whereas such compounds are no longer essential once the fruit reaches full maturity.

Interestingly, a proportionally greater accumulation of Ty derivatives compared to HTy derivatives occurs during the first weeks after fruit set compared to the rest of the development and ripening. The average proportion of Ty derivatives is highest at 1 WAF (25%), gradually decreasing to 19% by 8 WAF, and continues to decline during ripening, where Ty derivatives represent less than 8% of total Ty and HTy derivatives across all cultivars. Furthermore, cultivars associated with oils rich in phenolic compounds (“Dokkar”, “Menya”, and “Piñonera”; Table S2, Supporting Information) exhibited maximum fruit phenolic contents exceeding 190 μmol/g FW at 3 WAF, whereas cultivars producing oils with medium or low phenolic levels showed lower concentrations.

Regarding *TDC* gene expression, both *OeTDC1* and *OeTDC2* profiles largely mirrored the phenolic accumulation pattern during early fruit development, consistent with previous findings at later stages.^27,30^ As in those later stages, *OeTDC1* expression was constantly higher than *OeTDC2* (approximately 10-fold on average) though with significant cultivar-dependent variation. Overall, both genes exhibited a rapid increase in expression from anthesis (0 WAF), peaking within 1–3 WAF. However, except for “Abou kanani’”, the expression maxima of *OeTDC1* and *OeTDC2* were not synchronized, suggesting distinct regulatory mechanisms. For instance, *OeTDC1* peaked at 1 WAF in “Picual”, “Arbequina”, “Fishomi”, and “Menya”, and at 3 WAF in “Dokkar”, “Piñonera”, and “Abou kanani”, whereas *OeTDC2* showed the opposite trend, with the noted exception. Additionally, cultivars with an earlier *OeTDC1* peak often exhibited a secondary peak at 8 WAF, except for cultivar “Fishomi”.

Correlation analyses were performed between Ty and HTy derivative contents in fruits and *TDC* gene expression levels (or their sum) (Table 3). As in a previous study covering development from 3 WAF onward,^17^ Pearson correlation coefficients for the 0–3 WAF period were significant (*p* ≤ 0.05) and moderate-to-high across all comparisons between phenolic content and *OeTDC1*, *OeTDC2*, or their combined expression. Once again, correlations for Ty derivatives were consistently slightly higher than those for HTy derivatives, as had already been observed in later fruit development stages.^17^

**Table 3:** Pearson Correlation Coefficients (*r*) Between the Expression Levels of Olive *TDC* Genes and the Content of the Main Groups of Phenolics in the Fruit Early Development (0-3 WAF) of Seven Olive Cultivars with Contrasting Phenolics Contents (*p* ≤ 0.05)

	*OeTDC1*	*OeTDC2*	*OeTDC1* + *OeTDC2*
HTy-Derivatives	0.419	0.591	0.466
Ty-Derivatives	0.466	0.560	0.509
Ty + HTy-Derivatives	0.441	0.595	0.487

A strong correlation was previously reported between the phenolic compound content in olive fruits and their corresponding oils in a core collection from the World Olive Germplasm Bank,^9^ and more recently in a set of olive cultivars throughout fruit ripening.^27^ In this study, we investigated the possible role of olive *TDC* genes (*OeTDC1* and *OeTDC2*) in determining the phenolic content and profile during this process of fruit ripening. To this end, expression levels of *OeTDC1* and *OeTDC2* were quantified at different ripening stages (green, green-yellow, turning, and purple) and compared with the levels of Ty and HTy derivatives in the fruit (Figure 2 and Table 4).

**Figure 2:**
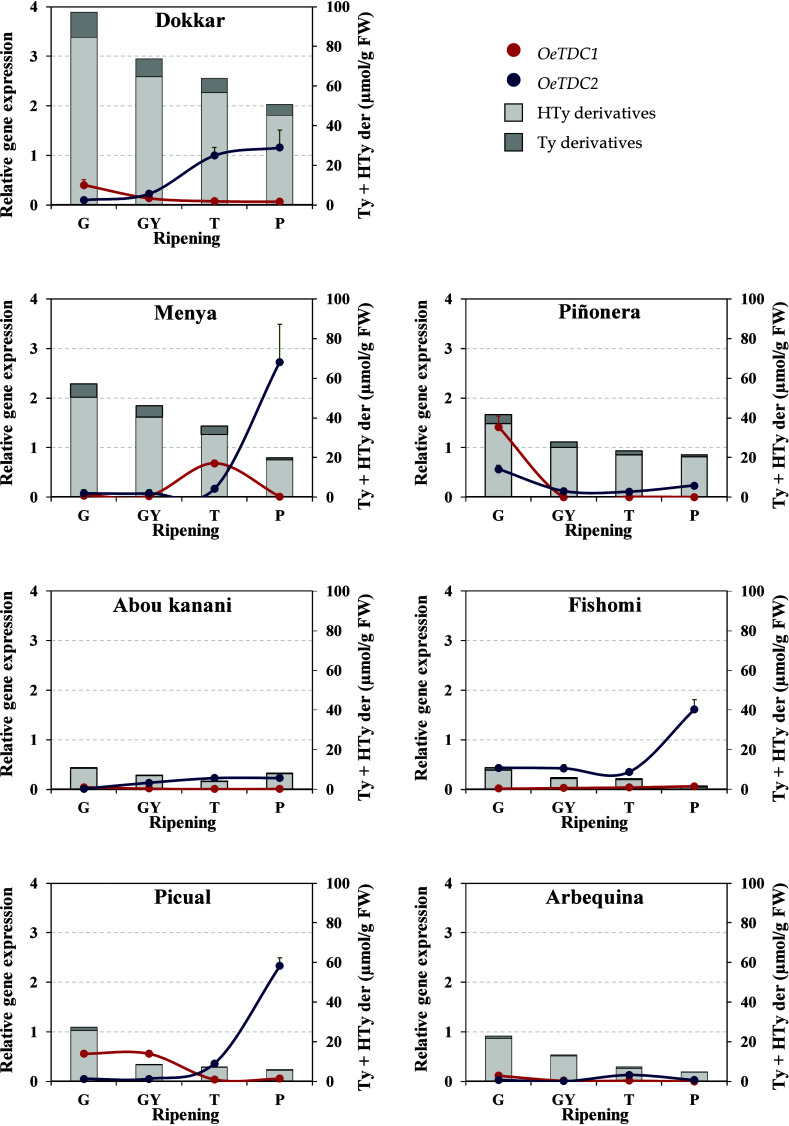
Relative expression levels of olive *TDC* genes in the mesocarp tissue of olive fruits along fruit ripening (G, dark green fruits; GY, green-yellow fruits; T, turning fruits with around 50% color; P, purple fruits, fully colored fruits with white mesocarp). Relative gene expression levels were scaled using as calibrator the expression of the *OeTDC2* gene in the “Dokkar” cultivar at stage T (control = 1). Data are mean ± SD of three biological and two technical replicates for each sample. Content of derivatives of tyrosol (Ty) and hydroxytyrosol (Hty) (μmol/g FW) were represented according to Table 4.

**Table 4:** Content (μmol/g FW) of the Main Classes of Phenolic Compounds (Hty-Der, Derivatives of Hydroxytyrosol; Ty-Der, Derivatives of Tyrosol; Total, Sum of Hty-Der and Ty-Der) in Olive Fruits of Seven Cultivars During Fruit Ripening, (G, Dark Green Fruits; GY, Green-Yellow Fruits; T, Turning Fruits with Around 50% Color; P, Purple Fruits, Fully Colored Fruits with White Mesocarp)^a^

Cultivar	Stage	HTy-Der	Ty-Der	Total
Dokkar	G	162.81 ± 12.12	25.83 ± 2.51	188.64 ± 14.62
	GY	126.43 ± 0.07	18.37 ± 1.00	144.80 ± 0.93
	T	112.43 ± 3.37	14.97 ± 1.43	127.40 ± 4.80
	P	86.62 ± 1.08	10.68 ± 0.00	97.30 ± 1.08
				
Menya	G	94.40 ± 3.28	13.05 ± 0.67	107.44 ± 3.95
	GY	75.90 ± 3.28	11.14 ± 0.67	87.04 ± 3.95
	T	60.14 ± 0.99	8.20 ± 0.72	68.35 ± 1.71
	P	36.98 ± 0.32	1.80 ± 0.01	38.79 ± 0.31
				
Piñonera	G	69.34 ± 3.91	8.85 ± 0.07	78.19 ± 3.98
	GY	47.34 ± 2.55	5.66 ± 0.22	53.00 ± 2.77
	T	40.23 ± 0.95	4.32 ± 0.01	44.54 ± 0.94
	P	40.05 ± 0.99	2.46 ± 0.04	42.51 ± 0.95
				
Abou kanani	G	20.00 ± 0.39	0.39 ± 0.17	20.39 ± 0.80
	GY	13.37 ± 0.30	0.30 ± 0.01	13.67 ± 0.18
	T	7.89 ± 0.24	0.24 ± 0.04	8.13 ± 2.36
	P	16.11 ± 0.35	0.35 ± 0.07	16.46 ± 1.25
				
Fishomi	G	18.97 ± 0.34	2.50 ± 0.10	21.47 ± 0.24
	GY	11.16 ± 0.95	1.29 ± 0.24	12.45 ± 1.19
	T	10.38 ± 0.68	1.09 ± 0.06	11.47 ± 0.62
	P	3.48 ± 0.08	0.87 ± 0.03	4.35 ± 0.12
				
Picual	G	48.47 ± 6.05	3.12 ± 0.75	51.60 ± 6.81
	GY	15.86 ± 0.47	0.82 ± 0.02	16.68 ± 0.45
	T	13.69 ± 0.27	0.68 ± 0.04	14.37 ± 0.22
	P	11.37 ± 0.05	0.73 ± 0.08	12.10 ± 0.13
				
Arbequina	G	40.72 ± 0.38	2.50 ± 0.33	43.22 ± 0.06
	GY	24.74 ± 1.11	0.82 ± 0.03	25.56 ± 1.15
	T	13.04 ± 0.41	1.35 ± 0.03	14.38 ± 0.38
	P	9.36 ± 0.29	0.08 ± 0.02	9.44 ± 0.31

a The content of the main individual phenolics can be found in the Supporting Information (Table S1).

As for these phenolics, a decline in their concentration was observed as ripening progressed, following the typical decrease that occurs during fruit development after the peak accumulation around 3–8 WAF previously documented in several studies.^27,30,31^ This reduction ultimately determines the Ty and HTy derivative contents in the oil. At the optimal oil extraction stage (T, turning), cultivars selected for producing oils with higher phenolic levels, particularly Ty and HTy derivatives, showed fruit concentrations above 40 μmol/g FW (“Dokkar”, “Menya”, and “Piñonera”), while medium-phenolic cultivars (“Picual” and “Arbequina”) exhibited values above 14 μmol/g FW, and phenolic-poor cultivars (“Abou kanani” and “Fishomi”) displayed lower levels.

Regarding the olive *TDC* genes presumably involved in Ty and HTy derivative biosynthesis, expression levels of *OeTDC1* and *OeTDC2* were found to be markedly lower (approximately 1200-fold and 16-fold, respectively) than the maximum expression detected during early fruit development. Similarly, Mougiou et al.^30^ reported a dramatic decrease in the abundance of the transcript annotated as *TDC* after ∼8 WAF in “Koroneiki” fruits. Unlike in fruit development, *OeTDC2* expression was generally higher than *OeTDC1* and showed a slight increase with ripening across all studied cultivars, with less pronounced differences in “Piñonera”, “Abou kanani”, and “Arbequina”. This expression trend for *OeTDC2* contrasts with the decline in the phenolic content during ripening (Table 4), suggesting that *OeTDC2* does not contribute to Ty or HTy derivative accumulation at this period and may instead be associated with other metabolic processes linked to fruit senescence. Likewise, *OeTDC1* appears to play a negligible role, given its very low expression levels. Recent evidence indicates that the limited synthesis and accumulation of Ty and HTy derivatives during ripening would be more closely related to *OeAAS* expression, a gene from the same family as TDCs but encoding a bifunctional enzyme with decarboxylation and oxidative deamination activity in a single step.^31^ Therefore, the final content of Ty and HTy derivatives in mature fruits primarily originates from residual phenolics accumulated during early development, along with possible contributions from *de novo* synthesis mediated by the OeAAS activity.

In this regard, a very high level of correlation was observed between the contents of HTy and Ty derivatives during the early stages of fruit development and the different stages of fruit ripening (Supporting Information, Table S3). The highest correlation coefficients (*r* > 0.955, *p* ≤ 0.001) were found for HTy and Ty derivatives levels in fruits at 8 WAF. Given the strong relationship also found between the phenolic content of the fruit and that of its oil (*r* > 0.904, *p* ≤ 0.001; Supporting Information, Table S4), as previously reported,^27,29^ it was therefore apparent that the phenolic composition during the early developmental stages also determines the phenolic content of the corresponding oils (Supporting Information, Table S5). Thus, the Pearson correlation coefficients found for the content of HTy and Ty derivatives in fruits with 8 WAF and the oils extracted from fruits at ripening stages GY, T and P were 0.942, 0.947, and 0.906 (*p* ≤ 0.01), respectively.

In summary, to gain deeper insight into early olive fruit growth and ripening in relation to the expression of *TDC* genes involved in the initial step of the biosynthesis of Ty and HTy, key components of the main phenolics in both fruit and oil, we analyzed the expression levels of *OeTDC1* and *OeTDC2*. Our results indicate that both *TDC* genes were strongly induced during the active cell division phase within the first 3 weeks after flowering, coinciding with a sharp increase in Ty and HTy concentrations. Notably, *OeTDC1* exhibited expression levels approximately ten times higher than *OeTDC2*, suggesting its predominant role in the first step of Ty and HTy biosynthesis in olive fruit and, ultimately, virgin olive oil. In contrast, neither gene participates in this synthesis during fruit ripening. Therefore, Ty and HTy derivatives present at this stage and consequently in the oil are largely remnants of their catabolism following the substantial accumulation that occurs early in development.

## Supplementary Material



## Notes

Content (μmol/g FW) of the main phenolics in olive fruits along early development and ripening, content (μmol/g oil) of the main phenolics in olive oils during ripening, content (μmol) per olive fruit of the main phenolics in olive fruits along early development and ripening, and Pearson correlation coefficients between the main phenolic groups in the fruit during early development and throughout ripening, in fruit and oil during ripening, and in fruits at 8 WAF and those in oils during ripening (PDF)
