# Supplementary material for: Early Developmental Regulation of Tyrosine Decarboxylase in Olive Fruits and Its Impact on Virgin Olive Oil Phenolics
Source: J Agric Food Chem. 2026 Apr 28;74(17):13951–8. doi: 10.1021/acs.jafc.5c17310 (PMC13154185; doi:10.1021/acs.jafc.5c17310)
Supplement: Supplementary file 1 [file jf5c17310_si_001.pdf]

Supporting Information

Early Developmental Regulation of Tyrosine Decarboxylase in Olive Fruits and Its Impact on Virgin Olive Oil Phenolics

Pilar Luaces, Rosario Sánchez, Jesús Expósito, Ana G. Pérez, Carlos Sanz*

Department of Biochemistry and Molecular Biology of Plant Products, Instituto de la Grasa, Spanish National Research Council (CSIC), 41013 Seville, Spain

Table S1. Content (µmol/g FW) of the main derivatives of Ty and HTy in olive fruits of seven cultivars. Sampling was performed by hand at full flowering (0 weeks after flowering, WAF) and up to 8 WAF. Additionally, fruit samples were taken during ripening at different stages (G, dark green fruits; GY, green-yellow fruits; T, turning fruits; P, purple fruits).
HTy-Der: derivatives of hydroxytyrosol; Ty-Der: derivatives of tyrosol; HTy+Ty-Der: sum of Hty-Der and Ty-Der.

	**HTy 1-glucoside**	**Verbascoside**	**Oleuropein**	**Oleuropein demethylated**	**Oleuropein aglycone**	**Ty 1-glucoside**	**Ligstroside**	**Ligstroside aglycone**	**HTy-Der**	**Ty-Der**	**HTy+Ty-Der**
Dokkar 0 WAF	6.59 ± 1.07	4.32 ± 0.43	32.52 ± 3.08	1.03 ± 0.12	0.08 ± 0.08	0.64 ± 0.04	1.02 ± 0.05	0.01 ± 0.00	44.54 ± 4.78	1.67 ± 0.09	46.22 ± 4.87
Dokkar 1 WAF	15.29 ± 0.25	2.19 ± 0.61	128.08 ± 13.06	0.16 ± 0.11	0.78 ± 0.71	11.04 ± 0.16	47.08 ± 4.20	0.24 ± 0.23	146.50 ± 13.30	58.37 ± 4.27	204.87 ± 17.57
Dokkar 3 WAF	13.57 ± 0.56	0.94 ± 0.12	188.59 ± 3.71	0.07 ± 0.03	0.15 ± 0.03	8.37 ± 0.07	51.96 ± 2.19	0.04 ± 0.00	203.32 ± 3.33	60.36 ± 2.26	263.69 ± 5.59
Dokkar 8 WAF	5.13 ± 0.14	3.91 ± 0.01	201.46 ± 3.80	0.02 ± 0.00	0.73 ± 0.03	4.55 ± 0.03	50.69 ± 1.03	0.33 ± 0.04	211.25 ± 3.70	55.57 ± 0.97	266.82 ± 4.66
Dokkar G	5.15 ± 0.25	8.09 ± 0.70	130.46 ± 10.01	0.06 ± 0.03	19.05 ± 1.62	0.50 ± 0.02	21.93 ± 2.30	3.41 ± 0.23	162.81 ± 12.12	25.83 ± 2.51	188.64 ± 14.62
Dokkar GY	4.06 ± 0.01	5.81 ± 0.06	96.57 ± 0.65	0.02 ± 0.01	19.96 ± 0.62	0.30 ± 0.02	14.98 ± 0.79	3.09 ± 0.19	126.43 ± 0.07	18.37 ± 1.00	144.80 ± 0.93
Dokkar T	4.07 ± 0.17	5.80 ± 0.23	76.82 ± 4.12	4.06 ± 0.13	21.69 ± 1.28	0.39 ± 0.04	11.46 ± 1.37	3.12 ± 0.02	112.43 ± 3.37	14.97 ± 1.43	127.40 ± 4.80
Dokkar P	5.09 ± 0.11	5.65 ± 0.10	10.86 ± 1.92	64.47 ± 1.06	0.54 ± 0.00	0.64 ± 0.02	9.98 ± 0.04	0.06 ± 0.06	86.62 ± 1.08	10.68 ± 0.00	97.30 ± 1.08
Menya 0 WAF	3.81 ± 0.11	3.92 ± 0.33	33.22 ± 0.08	1.78 ± 0.13	0.03 ± 0.01	0.36 ± 0.07	0.80 ± 0.04	0.01 ± 0.01	42.75 ± 0.65	1.18 ± 0.10	43.94 ± 0.55
Menya 1 WAF	7.73 ± 0.45	1.08 ± 0.18	153.98 ± 3.05	0.56 ± 0.43	0.70 ± 0.12	5.91 ± 0.12	39.37 ± 0.62	0.12 ± 0.03	164.04 ± 3.10	45.40 ± 0.71	209.44 ± 3.81
Menya 3 WAF	6.67 ± 0.47	0.00 ± 0.00	169.31 ± 10.36	0.31 ± 0.43	0.02 ± 0.01	4.33 ± 0.32	26.67 ± 3.23	0.03 ± 0.03	176.31 ± 10.40	31.03 ± 3.52	207.34 ± 13.92
Menya 8 WAF	4.67 ± 0.10	0.03 ± 0.00	146.57 ± 3.71	0.08 ± 0.00	1.36 ± 0.23	3.67 ± 0.13	31.13 ± 0.69	0.71 ± 0.07	152.71 ± 4.04	35.50 ± 0.90	188.22 ± 4.93
Menya G	2.30 ± 0.09	0.52 ± 0.04	91.15 ± 3.25	0.10 ± 0.01	0.33 ± 0.10	0.54 ± 0.05	12.21 ± 0.67	0.30 ± 0.04	94.40 ± 3.28	13.05 ± 0.67	107.44 ± 3.95
Menya GY	2.30 ± 0.09	0.52 ± 0.04	72.65 ± 3.25	0.10 ± 0.01	0.33 ± 0.10	0.54 ± 0.05	10.30 ± 0.67	0.30 ± 0.04	75.90 ± 3.28	11.14 ± 0.67	87.04 ± 3.95
Menya T	2.38 ± 0.20	1.01 ± 0.09	53.54 ± 0.46	1.13 ± 0.05	2.08 ± 0.29	0.27 ± 0.05	7.58 ± 0.66	0.34 ± 0.02	60.14 ± 0.99	8.20 ± 0.72	68.35 ± 1.71
Menya P	3.57 ± 0.08	0.84 ± 0.01	8.93 ± 0.53	23.41 ± 0.08	0.23 ± 0.07	0.35 ± 0.02	1.34 ± 0.05	0.11 ± 0.04	36.98 ± 0.32	1.80 ± 0.01	38.79 ± 0.31
Piñonera 0 WAF	8.36 ± 0.74	4.76 ± 1.36	32.92 ± 3.28	1.31 ± 0.20	0.04 ± 0.01	0.61 ± 0.15	0.68 ± 0.12	0.02 ± 0.01	47.38 ± 5.56	1.31 ± 0.26	48.69 ± 5.82
Piñonera 1 WAF	15.20 ± 1.32	0.09 ± 0.06	129.47 ± 1.71	0.26 ± 0.10	0.03 ± 0.02	7.78 ± 0.90	45.23 ± 6.00	0.03 ± 0.00	145.05 ± 0.33	53.04 ± 6.90	198.09 ± 7.23
Piñonera 3 WAF	9.07 ± 0.45	0.01 ± 0.01	149.16 ± 2.80	0.05 ± 0.04	0.05 ± 0.01	5.14 ± 0.16	34.68 ± 0.28	0.03 ± 0.04	158.33 ± 2.31	39.85 ± 0.48	198.18 ± 1.83
Piñonera 8 WAF	1.76 ± 0.18	0.80 ± 0.06	125.86 ± 2.79	0.05 ± 0.06	0.98 ± 0.08	3.38 ± 0.14	52.91 ± 0.13	0.74 ± 0.04	129.45 ± 2.92	57.03 ± 0.05	186.48 ± 2.97
Piñonera G	2.90 ± 0.24	3.83 ± 0.13	62.33 ± 3.57	0.19 ± 0.03	0.08 ± 0.05	0.76 ± 0.05	7.86 ± 0.05	0.23 ± 0.07	69.34 ± 3.91	8.85 ± 0.07	78.19 ± 3.98
Piñonera GY	3.75 ± 0.26	4.11 ± 0.43	37.61 ± 1.86	1.74 ± 0.02	0.13 ± 0.02	0.78 ± 0.06	4.87 ± 0.17	0.01 ± 0.00	47.34 ± 2.55	5.66 ± 0.22	53.00 ± 2.77
Piñonera T	2.67 ± 0.09	2.99 ± 0.17	30.34 ± 0.51	3.61 ± 0.18	0.61 ± 0.01	0.54 ± 0.01	3.71 ± 0.02	0.07 ± 0.01	40.23 ± 0.95	4.32 ± 0.01	44.54 ± 0.94
Piñonera P	6.12 ± 0.06	3.88 ± 0.01	8.24 ± 0.82	21.75 ± 0.15	0.06 ± 0.04	1.25 ± 0.02	1.14 ± 0.10	0.08 ± 0.04	40.05 ± 0.99	2.46 ± 0.04	42.51 ± 0.95
Abou kanani 0 WAF	7.42 ± 0.03	3.00 ± 0.39	56.72 ± 3.35	2.29 ± 0.48	0.05 ± 0.03	0.69 ± 0.13	2.61 ± 0.19	0.01 ± 0.00	69.48 ± 3.25	3.30 ± 0.32	72.78 ± 3.58
Abou kanani 1 WAF	7.99 ± 0.81	0.03 ± 0.04	114.66 ± 3.27	0.18 ± 0.05	2.22 ± 1.72	4.96 ± 0.04	25.34 ± 0.28	0.36 ± 0.26	125.08 ± 4.17	30.67 ± 0.50	155.75 ± 4.67
Abou kanani 3 WAF	6.93 ± 0.38	0.01 ± 0.00	134.56 ± 3.20	0.03 ± 0.04	0.03 ± 0.00	4.72 ± 0.17	27.38 ± 0.03	0.05 ± 0.05	141.55 ± 3.54	32.15 ± 0.10	173.70 ± 3.45
Abou kanani 8 WAF	2.02 ± 0.03	0.07 ± 0.01	110.89 ± 0.64	0.03 ± 0.01	1.61 ± 0.03	1.65 ± 0.03	17.44 ± 0.07	0.64 ± 0.02	114.62 ± 0.60	19.73 ± 0.07	134.35 ± 0.67
Abou kanani G	0.88 ± 0.05	0.04 ± 0.00	18.92 ± 0.57	0.12 ± 0.01	0.04 ± 0.00	0.01 ± 0.01	0.37 ± 0.17	0.01 ± 0.00	20.00 ± 0.62	0.39 ± 0.17	20.39 ± 0.80
Abou kanani GY	0.82 ± 0.02	0.09 ± 0.00	12.27 ± 0.24	0.05 ± 0.06	0.15 ± 0.01	0.02 ± 0.00	0.28 ± 0.01	0.01 ± 0.00	13.37 ± 0.19	0.30 ± 0.01	13.67 ± 0.18
Abou kanani T	0.99 ± 0.48	0.15 ± 0.07	6.56 ± 1.90	0.04 ± 0.05	0.14 ± 0.17	0.02 ± 0.01	0.16 ± 0.07	0.06 ± 0.03	7.89 ± 2.32	0.24 ± 0.04	8.13 ± 2.36
Abou kanani P	4.16 ± 0.30	0.52 ± 0.02	11.42 ± 0.87	0.01 ± 0.01	0.01 ± 0.01	0.03 ± 0.00	0.23 ± 0.02	0.09 ± 0.06	16.11 ± 1.17	0.35 ± 0.07	16.46 ± 1.25
Fishomi 0 WAF	2.72 ± 0.55	3.33 ± 0.35	35.12 ± 4.39	0.66 ± 0.19	0.02 ± 0.00	0.57 ± 0.00	2.77 ± 0.16	0.03 ± 0.00	41.86 ± 5.49	3.38 ± 0.17	45.23 ± 5.66
Fishomi 1 WAF	1.64 ± 0.02	0.03 ± 0.00	117.39 ± 12.16	0.62 ± 0.48	0.13 ± 0.09	1.61 ± 0.41	38.26 ± 5.61	0.02 ± 0.01	119.81 ± 12.76	39.89 ± 6.03	159.70 ± 18.80
Fishomi 3 WAF	2.58 ± 0.03	0.00 ± 0.00	111.77 ± 0.58	0.07 ± 0.02	0.02 ± 0.01	1.92 ± 0.10	20.80 ± 0.02	0.02 ± 0.00	114.45 ± 0.58	22.74 ± 0.08	137.18 ± 0.50
Fishomi 8 WAF	0.70 ± 0.02	0.00 ± 0.00	75.90 ± 1.87	0.07 ± 0.01	1.50 ± 0.06	0.83 ± 0.03	10.93 ± 0.17	0.35 ± 0.01	78.16 ± 1.92	12.11 ± 0.15	90.27 ± 2.07
Fishomi G	2.35 ± 0.04	0.41 ± 0.01	16.07 ± 0.34	0.11 ± 0.00	0.03 ± 0.03	0.20 ± 0.01	2.28 ± 0.11	0.01 ± 0.00	18.97 ± 0.34	2.50 ± 0.10	21.47 ± 0.24
Fishomi GY	3.00 ± 0.57	0.17 ± 0.02	7.81 ± 0.56	0.05 ± 0.04	0.13 ± 0.16	0.21 ± 0.05	1.07 ± 0.20	0.02 ± 0.00	11.16 ± 0.95	1.29 ± 0.24	12.45 ± 1.19
Fishomi T	2.87 ± 0.07	0.06 ± 0.01	7.42 ± 0.63	0.01 ± 0.00	0.02 ± 0.00	0.21 ± 0.01	0.81 ± 0.02	0.06 ± 0.08	10.38 ± 0.68	1.09 ± 0.06	11.47 ± 0.62
Fishomi P	2.86 ± 0.05	0.23 ± 0.01	0.28 ± 0.04	0.06 ± 0.06	0.06 ± 0.01	0.13 ± 0.01	0.70 ± 0.03	0.04 ± 0.00	3.48 ± 0.08	0.87 ± 0.03	4.35 ± 0.12
Picual 0 WAF	3.63 ± 0.51	2.99 ± 0.64	34.29 ± 3.14	0.59 ± 0.19	0.06 ± 0.00	0.67 ± 0.05	1.34 ± 0.03	0.02 ± 0.02	41.56 ± 4.09	2.03 ± 0.00	43.58 ± 4.09
Picual 1 WAF	4.17 ± 0.07	0.10 ± 0.03	135.98 ± 8.43	0.17 ± 0.04	0.14 ± 0.09	6.72 ± 0.02	53.96 ± 0.45	0.05 ± 0.01	140.56 ± 8.47	60.72 ± 0.44	201.28 ± 8.04
Picual 3 WAF	2.79 ± 0.35	0.01 ± 0.01	139.62 ± 10.18	0.11 ± 0.02	0.02 ± 0.01	3.00 ± 0.19	33.40 ± 2.88	0.03 ± 0.01	142.54 ± 9.82	36.42 ± 3.07	178.97 ± 12.89
Picual 8 WAF	2.18 ± 0.05	0.00 ± 0.00	84.13 ± 2.94	0.29 ± 0.06	1.91 ± 0.20	2.91 ± 0.01	21.81 ± 0.32	0.80 ± 0.06	88.51 ± 2.84	25.52 ± 0.27	114.03 ± 3.11
Picual G	2.41 ± 0.49	1.51 ± 0.31	44.14 ± 5.18	0.39 ± 0.10	0.03 ± 0.03	0.21 ± 0.08	2.86 ± 0.73	0.05 ± 0.05	48.47 ± 6.05	3.12 ± 0.75	51.60 ± 6.81
Picual GY	1.26 ± 0.11	0.53 ± 0.03	13.99 ± 0.32	0.01 ± 0.01	0.07 ± 0.00	0.03 ± 0.01	0.72 ± 0.01	0.07 ± 0.01	15.86 ± 0.47	0.82 ± 0.02	16.68 ± 0.45
Picual T	1.56 ± 0.12	0.55 ± 0.06	11.44 ± 0.31	0.09 ± 0.13	0.05 ± 0.00	0.11 ± 0.02	0.55 ± 0.03	0.02 ± 0.00	13.69 ± 0.27	0.68 ± 0.04	14.37 ± 0.22
Picual P	2.06 ± 0.04	0.91 ± 0.03	8.28 ± 0.09	0.09 ± 0.02	0.03 ± 0.01	0.16 ± 0.01	0.55 ± 0.09	0.02 ± 0.00	11.37 ± 0.05	0.73 ± 0.08	12.10 ± 0.13
Arbequina 0 WAF	3.01 ± 0.79	4.41 ± 0.07	36.21 ± 3.82	3.38 ± 0.13	0.02 ± 0.01	0.53 ± 0.51	1.22 ± 0.25	0.04 ± 0.01	47.02 ± 4.66	1.79 ± 0.75	48.81 ± 5.41
Arbequina 1 WAF	3.63 ± 1.29	0.51 ± 0.12	122.47 ± 1.13	0.52 ± 0.22	0.90 ± 0.57	3.54 ± 0.07	39.51 ± 3.24	0.18 ± 0.11	128.02 ± 1.95	43.23 ± 3.42	171.26 ± 1.46
Arbequina 3 WAF	3.47 ± 0.28	0.01 ± 0.00	143.59 ± 8.38	0.15 ± 0.01	0.01 ± 0.00	3.29 ± 0.10	25.83 ± 2.00	0.02 ± 0.01	147.24 ± 8.11	29.14 ± 2.11	176.38 ± 10.22
Arbequina 8 WAF	3.95 ± 0.00	0.43 ± 0.02	106.04 ± 2.07	0.07 ± 0.06	0.64 ± 0.01	2.24 ± 0.14	11.24 ± 0.21	0.21 ± 0.03	111.13 ± 2.14	13.69 ± 0.38	124.82 ± 2.53
Arbequina G	2.50 ± 0.04	3.25 ± 0.33	31.62 ± 0.17	3.31 ± 0.13	0.04 ± 0.03	0.07 ± 0.00	2.40 ± 0.34	0.03 ± 0.02	40.72 ± 0.38	2.50 ± 0.33	43.22 ± 0.06
Arbequina GY	1.90 ± 0.13	1.65 ± 0.07	6.59 ± 0.01	14.51 ± 0.93	0.09 ± 0.00	0.01 ± 0.00	0.80 ± 0.03	0.01 ± 0.01	24.74 ± 1.11	0.82 ± 0.03	25.56 ± 1.15
Arbequina T	1.59 ± 0.02	0.91 ± 0.04	0.01 ± 0.00	10.45 ± 0.40	0.07 ± 0.00	0.02 ± 0.00	1.30 ± 0.03	0.03 ± 0.00	13.04 ± 0.41	1.35 ± 0.03	14.38 ± 0.38
Arbequina P	1.56 ± 0.07	0.90 ± 0.01	0.04 ± 0.03	6.80 ± 0.40	0.05 ± 0.00	0.04 ± 0.01	0.02 ± 0.00	0.02 ± 0.01	9.36 ± 0.29	0.08 ± 0.02	9.44 ± 0.31

Table S2. Content (µmol/g oil) of the main derivatives of Ty and HTy in olive oils of seven cultivars during ripening (G, dark green fruits; GY, green-yellow fruits; T, turning fruits; P, purple fruits). HTy-Der: derivatives of hydroxytyrosol; Ty-Der: derivatives of tyrosol; HTy+Ty-Der: sum of Hty-Der and Ty-Der

	**HTy**	**HTy acetate**	**Oleacein**	**Oleuropein aglycone**	**Ty**	**Oleocanthal**	**Ligstroside aglycone**	**HTy-Der**	**Ty-Der**	**HTy+Ty-Der**
Dokkar GY	0.072 ± 0.003	0.005 ± 0.001	0.150 ± 0.000	2.500 ± 0.031	0.053 ± 0.004	0.120 ± 0.066	2.382 ± 0.045	2.728 ± 0.033	2.554 ± 0.115	5.282 ± 0.148
Dokkar T	0.085 ± 0.004	0.005 ± 0.000	0.325 ± 0.011	2.191 ± 0.135	0.053 ± 0.002	0.368 ± 0.005	1.804 ± 0.040	2.606 ± 0.150	2.226 ± 0.047	4.832 ± 0.197
Dokkar P	0.026 ± 0.000	0.052 ± 0.001	2.084 ± 0.117	0.817 ± 0.070	0.021 ± 0.001	1.708 ± 0.008	0.462 ± 0.013	2.980 ± 0.047	2.192 ± 0.023	5.171 ± 0.070
Menya GY	0.041 ± 0.011	0.006 ± 0.002	0.023 ± 0.002	1.267 ± 0.003	0.109 ± 0.009	0.157 ± 0.003	1.245 ± 0.048	1.336 ± 0.017	1.512 ± 0.042	2.848 ± 0.024
Menya T	0.081 ± 0.014	0.008 ± 0.001	0.120 ± 0.012	0.561 ± 0.058	0.100 ± 0.015	0.214 ± 0.030	0.771 ± 0.052	0.770 ± 0.055	1.085 ± 0.068	1.855 ± 0.122
Menya P	0.047 ± 0.001	0.063 ± 0.001	0.284 ± 0.001	0.252 ± 0.009	0.084 ± 0.004	0.362 ± 0.006	0.276 ± 0.000	0.645 ± 0.008	0.722 ± 0.010	1.367 ± 0.002
Piñonera GY	0.009 ± 0.001	0.015 ± 0.001	0.643 ± 0.001	0.104 ± 0.002	0.048 ± 0.002	0.747 ± 0.015	0.102 ± 0.025	0.772 ± 0.002	0.898 ± 0.041	1.670 ± 0.044
Piñonera T	0.010 ± 0.000	0.011 ± 0.001	0.646 ± 0.022	0.108 ± 0.001	0.058 ± 0.000	0.808 ± 0.025	0.085 ± 0.007	0.775 ± 0.022	0.952 ± 0.018	1.727 ± 0.040
Piñonera P	0.023 ± 0.001	0.042 ± 0.003	0.811 ± 0.017	0.072 ± 0.008	0.106 ± 0.003	0.582 ± 0.009	0.054 ± 0.009	0.949 ± 0.029	0.741 ± 0.021	1.690 ± 0.050
Abou kanani GY	0.043 ± 0.002	0.028 ± 0.004	0.073 ± 0.013	0.187 ± 0.021	0.021 ± 0.002	0.039 ± 0.002	0.015 ± 0.001	0.331 ± 0.010	0.075 ± 0.002	0.406 ± 0.008
Abou kanani T	0.026 ± 0.003	0.024 ± 0.001	0.070 ± 0.002	0.170 ± 0.012	0.012 ± 0.001	0.028 ± 0.003	0.007 ± 0.000	0.289 ± 0.018	0.048 ± 0.005	0.337 ± 0.023
Abou kanani P	0.013 ± 0.000	0.023 ± 0.000	0.018 ± 0.000	0.048 ± 0.000	0.006 ± 0.000	0.008 ± 0.000	0.004 ± 0.000	0.103 ± 0.000	0.017 ± 0.000	0.120 ± 0.000
Fishomi GY	0.019 ± 0.002	0.015 ± 0.002	0.098 ± 0.013	0.128 ± 0.011	0.041 ± 0.001	0.087 ± 0.008	0.094 ± 0.003	0.261 ± 0.027	0.222 ± 0.011	0.484 ± 0.039
Fishomi T	0.020 ± 0.000	0.011 ± 0.000	0.069 ± 0.004	0.139 ± 0.006	0.037 ± 0.001	0.070 ± 0.004	0.106 ± 0.003	0.239 ± 0.009	0.213 ± 0.000	0.451 ± 0.009
Fishomi P	0.021 ± 0.000	0.007 ± 0.000	0.058 ± 0.003	0.304 ± 0.009	0.031 ± 0.001	0.050 ± 0.003	0.136 ± 0.011	0.390 ± 0.013	0.217 ± 0.015	0.607 ± 0.028
Picual GY	0.009 ± 0.000	0.013 ± 0.000	0.136 ± 0.004	0.420 ± 0.001	0.009 ± 0.001	0.090 ± 0.003	0.260 ± 0.023	0.578 ± 0.005	0.359 ± 0.022	0.937 ± 0.017
Picual T	0.008 ± 0.000	0.011 ± 0.001	0.059 ± 0.000	0.284 ± 0.000	0.017 ± 0.001	0.053 ± 0.001	0.214 ± 0.009	0.363 ± 0.002	0.284 ± 0.009	0.647 ± 0.011
Picual P	0.014 ± 0.000	0.010 ± 0.000	0.051 ± 0.000	0.270 ± 0.001	0.034 ± 0.000	0.039 ± 0.001	0.172 ± 0.013	0.345 ± 0.001	0.244 ± 0.012	0.590 ± 0.014
Arbequina GY	0.008 ± 0.000	0.053 ± 0.000	0.410 ± 0.011	0.046 ± 0.004	0.011 ± 0.000	0.271 ± 0.007	0.004 ± 0.000	0.517 ± 0.007	0.287 ± 0.007	0.804 ± 0.014
Arbequina T	0.012 ± 0.000	0.112 ± 0.002	0.308 ± 0.005	0.032 ± 0.006	0.011 ± 0.001	0.178 ± 0.005	0.002 ± 0.001	0.464 ± 0.013	0.191 ± 0.007	0.655 ± 0.019
Arbequina P	0.004 ± 0.000	0.061 ± 0.000	0.083 ± 0.001	0.013 ± 0.002	0.008 ± 0.000	0.083 ± 0.002	0.002 ± 0.000	0.160 ± 0.003	0.093 ± 0.002	0.253 ± 0.005

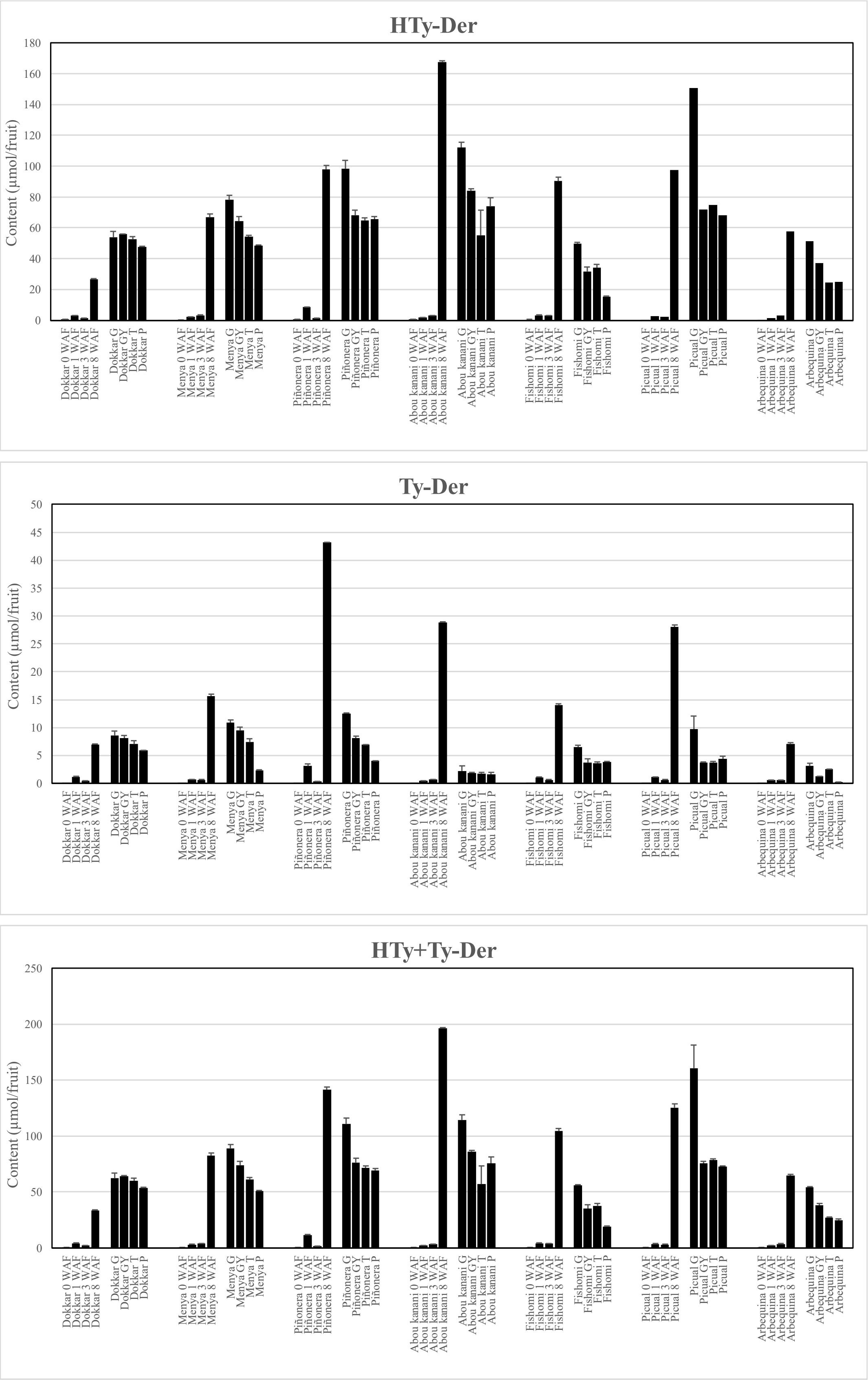

Figure S1. Content (μmol) per olive fruit of the main derivatives of Ty and HTy in seven cultivars during early development [from 0 to 8 weeks after flowering (WAF)] and ripening (G, dark green fruits; GY, green-yellow fruits; T, turning fruits; P, purple fruits).

Table S3. Pearson correlation coefficients (*r*) between the contents of hydroxytyrosol and tyrosol derivatives in the fruit during early development (0-8 WAF) and throughout ripening (GY, T and P stages) (** $p \leq 0.01$; *** $p \leq 0.001$).

	Green-Yellow (GY)	Turning (T)	Purple (P)
0 WAF (flower)	-0.323	-0.332	-0.184
1 WAF	0.683	0.678	0.618
3 WAF	0.938**	0.934**	0.953***
8 WAF	0.959***	0.955***	0.978***

Table S4. Pearson correlation coefficients (*r*) between the contents of the main phenolic groups in fruit and oil during ripening (GY, T and P stages) ($p \leq 0.001$).

	Oil - HTy-Der	Oil - Ty-Der	Oil – HTy+Ty Der
Fruit - HTy-Der	0.930	0.982	0.963
Fruit - Ty-Der	0.904	0.966	0.942
Fruit - HTy+Ty Der	0.929	0.982	0.963

Table S5. Pearson correlation coefficients (*r*) between the content of the main phenolic groups in fruits at 8 WAF and those in oils during ripening (GY, T and P stages) (* $p \leq 0.05$; ** $p \leq 0.01$).

	Green-Yellow (GY)	Turning (T)	Purple (P)
HTy-Der	0.950**	0.928**	0.874*
Ty-Der	0.778*	0.837*	0.796*
HTy+Ty Der	0.942**	0.947**	0.906**
